# Precordial ECG Lead Mispositioning: Its Incidence and Estimated Cost to Healthcare

**DOI:** 10.7759/cureus.9040

**Published:** 2020-07-07

**Authors:** Mahin Rehman, Najeeb U Rehman

**Affiliations:** 1 Internal Medicine, Guthrie Clinic, Robert Packer Hospital, Sayre, USA; 2 Cardiology, Guthrie Clinic, Robert Packer Hospital, Sayre, USA

**Keywords:** ecg, ekg, electrocardiography, cardiology, cardiology devices, cardiovascular disease, lead misplacement, cardiovascular education, healthcare cost, economic burden of healthcare

## Abstract

Background: The study was performed to estimate the incidence and economic burden of electrocardiogram (ECG) precordial lead mispositioning, in an effort to highlight the need for quality improvement. Lead mispositioning may result in further cardiovascular testing to rule out significant cardiac disease, thus adding to the national healthcare financial burden.

Methods: All consecutive adult ECGs done during 2018, were reviewed. ECGs with acute anterior myocardial infarction (AMI), bundle branch blocks, left ventricular hypertrophy (LVH), left anterior fascicular block (LAFB), pre-excitation, left axis deviation, ventricular pacing and low voltage QRS were excluded. Septal infarcts identified automatically by the computerized software or identified manually using the criteria of QS composite in V2 were not excluded. Computer interpreted ECGs as “cannot rule-out anterior infarct” were also not excluded from this data. Reimbursement of various stress test types was used to estimate the cost burden of misdiagnosed ECGs.

Results: A total of 9424 adult ECGs were evaluated. Poor R-wave progression (PRWP) or reversed R-wave progression (RRWP) accounted for 497 (5.27%) and 102 (1.08%) ECGs, respectively. A total of 335 septal infarct interpretations constituted about 3.55% of all ECGs. ECGs categorized as “cannot rule-out AMI” due to PRWP constituted about 0.89%. Therefore, a total of 1018 ECGs (10.8%) could be possibly falsely labelled as some type of myocardial infarction.

Conclusion: Precordial ECG lead mispositioning can lead to significantly abnormal ECG patterns, leading to false diagnoses and further unnecessary cardiovascular testing. This not only increases risk and cost to the patient, but also adds to the national healthcare financial burden.

## Introduction

The heartbeat is a mechanical function of the heart triggered by an electrical impulse. This electrical impulse consists of depolarization and repolarization signals resulting in cardiac systole and diastole. An electrocardiogram (ECG) is a graphical recording of the magnitude and direction of the electrical current generated during these depolarization and repolarization phases. The surface electrode positioning of the 12 lead ECG was standardized by the committee of the American Heart Association in 1938 and 1943 [1,2].

Competence in the recording and interpretation of an ECG is required by the certifying bodies for physicians. Competency in ECG recording is also a requirement for ECG technicians, emergency medical technicians, and many nursing staff in critical clinical departments. The process of recording an ECG involves preparing the patient and their skin, accurate electrode placement, and checking the electrocardiograph settings and other equipment required for the procedure. ECGs can be affected by several technical and clinical factors which may then affect its interpretation and, thus, patient outcomes. The most common technical factors for surface electrode mispositioning are carelessness or haste, difficulty in identification of landmarks and, rarely, change of cardiac position [3,4]. Correct chest lead placement is essential for an accurate ECG recording and its interpretation. Commonly precordial electrodes are placed either too high or too low vertically and/or are horizontally displaced as well from their normal anatomically defined locations [5]. These lead mispositioning errors result in erroneous interpretation of ECGs [6].

In clinical experience, chest lead placement is often inaccurate, and most common errors relate to the placement of leads V1 and V2 [7,8]. One of the most common chest lead placement error results in poor R-wave progression (PRWP) or reversed R-wave progression (RRWP), which are often interpreted as acute anterior myocardial infarction (AMI) [9]. In clinical practice, most of these interpretations will result in cardiology consultations and/or stress testing. Some of these patients, with no coronary heart disease, will have false positive stress tests and may go on to even having coronary angiography. 

In addition to AMI and lead mispositioning, PRWP or RRWP may also appear in the presence of incomplete or complete left bundle branch block (LBBB), right bundle branch block (RBBB), left ventricular hypertrophy (LVH), left anterior fascicular block (LAFB), pre-excitation, pseudo-Q-wave caused by perpendicular orientation of the initial QRS deflection to the lead axis, mitral valve prolapse, and abnormally low diaphragm position in pulmonary emphysema [10]. Less common causes of PRWP include spontaneous pneumothorax, dextrocardia, “corrected” transposition of the great vessels, and congenital absence of the pericardium [11].

In the past, a lot of effort has been made to increase the accuracy and positive predictive value of PRWP for diagnosing AMI. PRWP is common in patients with AMI. Therefore, AMI and coronary artery disease (CAD) is commonly expected in patients with ECGs having PRWP [12]. PRWP, RRWP and septal infarct can all result in a presumptive diagnosis of AMI or “cannot rule out AMI”. Since several ECG patterns can potentially be suspected for AMI and underlying CAD, and can result from erroneous lead mispositioning, there is a significant concern of healthcare dollars being wasted on inappropriate downstream cardiovascular stress testing to rule out clinically obstructive CAD. After conducting a search on PubMed, there is no significant data available in the context of the economic burden of this erroneous lead mispositioning. In this study, therefore, the main focus is simply on assessing the prevalence of precordial lead ECG patterns that could be interpreted and related to underlying CAD in outpatient settings so as to roughly estimate the economic burden of inappropriate downstream testing.

## Materials and methods

The resting ECGs of consecutive adult patients during the year 2018, at the outpatient Guthrie clinic, were retrieved using GE Muse™ 8.0.1 Cardiology Information System (Milwaukee, WI, USA) and analyzed retrospectively. Standard 12-lead ECGs were recorded in the supine position, by the ECG technicians, using recommended standardized procedures and MAC™ 5500HD electrocardiograph recorder version v-010B by GE Healthcare (Life Care Solutions, Milwaukee, WI, USA). ECG was recorded and printed using the standard paper speed of 50 mm/sec. The standard filter setting of the system (150 Hz) was used. Because of the common and worldwide use of the GE Marquette™ 12SL™ ECG analysis program, it was used for computerized analysis and interpretation of the ECGs, which were also then over-read and confirmed by a cardiologist.

We used the GE Marquette™ Muse system's criteria for first line auto-analysis and then the commonly used Young’s criteria of RV3 ≤2 mm was employed for secondary manual analysis [13,14]. Subjects with AMI, bundle branch blocks, LVH, LAFB, ventricular pacing and low voltage QRS were excluded. AMI was defined as Q-wave or QS composite in lead V3, V2 & V3 or V2-V6, or PRWP in precordial leads (V2-V6). For PRWP, several criteria have been developed to diagnose PRWP and are listed in Table 1. Low voltage ECG was defined as QRS amplitude of <5 mm in all limb leads or <10 mm in all precordial leads. ECGs with left axis deviation were also excluded, as it can be caused by LAFB as well as by LVH, LBBB, inferior infarct, ventricular pacing and Wolf-Parkinson-White (WPW) syndrome. Septal infarcts identified automatically by the computerized software or identified manually using the criteria of QS composite in V2 were not excluded, assuming that some of them may be artifactual due to lead mispositioning. Based upon the same reasoning, EKGs interpreted as “cannot rule-out anterior infarct” were not excluded from this data.

**Table 1:** Criteria for poor R-wave progression RV: R-wave amplitude in lead V (number). SV: S-wave amplitude in lead V (number).

Study	Poor R-Wave Progression Criteria
Young et al. [13]	RV3 ≤2 mm and/or RV4 ≤4 mm
Marquette system [14]	1) No left ventricular hypertrophy 2) RV3 or RV4 <2 mm and (a decrease in RV2 to RV3, or RV3 to RV4) or 3) RV3 <1 mm and (<0.25 mm increase from RV2 to RV3)
Zema et al. [15]	1) Decrease in RV1 to RV2, or RV2 to RV3, or RV3 to RV4 or RV4 ≤3 mm or RV3 ≤3 mm and RV2 ≤RV3 and 2) RV1 ≤4 mm and SV1 <1 mm or 3) RV1 >4 mm and (RV3 ≤1.5 mm or RV3 >1.5 mm with T wave inversion or ST elevation in V2 or V3)
DePace et al. [16]	1) RV3 ≤3 mm or (decrease in RV1 to RV2, and RV2 to RV3, and RV3 to RV4) and 2) (0.95a+1.38b+0.17c-0.12d-0.07e-0.54)>0, where a) 1 for men, 2 for women; b) 2 for ST depression or T wave inversion in both leads V2 and V3, 1 for ST depression or T wave inversion in V2 or V3, 0 for normal ST segments and T waves in V2 and V3; c) SV2; d) SV3; e) Sum of RV3 and V4
Warner et al. [17]	1) No left or right ventricular hypertrophy and 2) Duration of RV2 <20 millisecond

## Results

During 2018, there were a total of 9,424 ECG records available from 6,417 adult patients who had their ECG done as outpatient in one of the designated cardiology clinics. These ECGs were reviewed on the GE Muse™ 8.0.1 Cardiology Information System (Milwaukee, WI, USA). Out of these, 2,616 ECGs were excluded based upon the criteria discussed above. The total eligible ECGs, after exclusion, for evaluation were therefore 6,808. All the ECG interpretations and their incidence are detailed (Table 2). A total of 1,018 ECGs (10.8%) could be possibly labelled as some type of myocardial infarction suggesting underlying CAD. Of these total 1,018 subjects, 534 were female (52.5%) and 494 were male (48.5%).

**Table 2:** Electrocardiogram (ECG) interpretations PRWP: Poor R-wave progression; RRWP: Reverse R-wave progression.

Diagnoses	Number	% of Eligible ECGs	% of Total ECGs
PRWP	497	7.3	5.27
RRWP	102	1.49	1.08
Septal infarct (+ RV3 ≤2)	65	0.95	0.69
Septal infarct (+ RV3 >2)	270	3.96	2.86
Cannot rule-out AMI	84	1.23	0.89
All above diagnoses	1018	14.95	10.8

## Discussion

PRWP was the sixth most common abnormal ECG pattern in a consecutive series of 19,734 ECGs collected by the Metropolitan Life Insurance Company over a period of 5 ¼ years [18]. Some studies suggest that 50% or more ECGs have lead misplacement errors which affects their analysis and interpretation [7,19,20]. However, PRWP incidence is reported in some studies as listed in Table 3. A study from the National Cancer Center of Korea reported a lower prevalence of PRWP ranging between 0.5% to 1.8%, depending upon the two criteria [21]. PRWP had been reported to be in the range of 15%-42% based upon different criteria [22]. Yet, another study reported PRWP to be present in 137 patients out of 660 hospitalized patients making the prevalence of PRWP to be about 20.7% [23]. Total average incidence of PRWP from these six studies ranges from 11.37% to 16.08% (Table 3).

**Table 3:** Incidence of poor R-wave progression (PRWP) in various studies

PRWP Incidence	Studies
8%	DePace et al. [16]
0.5% - 1.8%	Kim et al. [21]
15%-42%	Gami et al. [22]
20.7%	Prajapat et al. [23]
10%	Zema et al. [24]
14% (19% ♀, 11% ♂)	Colaco et al. [25]
11.37% – 16.08%	Total Average

In a web search, average current national gross charge for a cardiac stress test is about $4,400 with a range of $1,200 to $11,700 [26]. On another health cost comparator website, Medicare allowed global reimbursement in 2018 at Robert Packer Hospital, Sayre, PA, is reportedly $1,183.10 and $531.86 for a nuclear stress test (multiple studies) and transthoracic stress echocardiogram (SE) with ECG recording, respectively [27]. As reimbursement of any test varies by the insurance plan, facility type (hospital vs. office) and geographic location, it is beyond the scope of this study to calculate one precise cost of the stress testing in the US. Therefore, just to understand the scope of our issue at hand, we will simply use our local Medicare allowable global reimbursement rates for hospital facility of $531.86 for a SE and of $1,183.10 for a nuclear stress test as the average cost. 

Cardiac stress testing is more commonly performed as nuclear stress imaging than stress-echocardiography. At Robert Packer Hospital, Sayre, PA, the stress tests done are grossly 30% SE and 70% nuclear stress tests. Given a total of 9,424 ECGs done annually at one specific Guthrie cardiology out-patient clinic, Sayre, PA, with an exclusion rate of about 27.75%, we had a total 6,808 ECGs evaluated with an eligibility rate of 72.25%. Precordial lead misplacement is suspected in 1,018 ECGs which have an abnormal pattern suggestive of myocardial infarction and, thus, possible underlying CAD. This constitutes 14.95% of the eligible ECGs (with exclusion criteria) or 10.8% of all the ECGs (without any exclusion). If all these patients with so-called abnormal ECGs and false myocardial infarctions were to undergo some sort of cardiac stress testing, we would approximately expect 305 patients to have SE and 713 patients to undergo a nuclear stress test. Using 2018 Medicare allowable reimbursement rates, as discussed above, the total cost of these tests from, solely, our facility to the healthcare system is about $1,005,768. 

To estimate similar cost to healthcare system in the US, there is no robust data available regarding annual total number of ECGs done in the United States (US), on PubMed or general web search. In one non-verifiable online report, the estimated number of annual ECGs done in the US was about 300 million in 2008 [28]. However, the Centers for Medicare & Medicaid Services (CMS) data from 2015 reported 18,332,027 office-based ECGs and 12,314,797 hospital/facility-based ECGs with a total number of 30,646,824 ECGs [29]. Using the CMS data of 30 million ECGs, expected cost to the CMS of ECG lead mispositioning based on our criteria discussed above, at gross incidence rate of 10.8%, would be about $3,201,069,077 ($3.2 billion).

Limitations

There is lack of a standard diagnostic criterion for PRWP. Several existing criteria are too complex to be routinely applied in clinical practice and thus remain confusing and challenging for general clinicians (Table 1). The Marquette system is widely available, commonly used and easily applicable as well, so it was used as first line criteria in our computerized interpretation system. As alluded to above, the cost of stress testing varies significantly across the US, and very gross estimates of the stress testing reimbursement at our facility are used to estimate our local and national costs to the healthcare system. Medicare allowable reimbursement data is used for cost analysis of all adult ECGs across the board. It is to be, therefore, understood that due to many of the generalizations, approximations and gross estimates used here, would all contribute to significant limitations of this study. 

Secondly, this analysis was also limited to the Guthrie Clinic; thus, being a single center is a limitation of this study. Without including other centers in our data set, generalizing does become difficult since lead application is clearly operator dependent and each institution’s departmental protocols, training procedures, and resources will all vary to a degree. 

Thirdly, another major limitation to consider is operatory dependency. Placement of ECG leads on a patient is operator dependent so accurate and appropriate placement will vary from individual to individual. Thus, the varying placement of ECG leads will lead to some degree of discrepancy of the ECG tracing, which could end up resulting in erroneous readings leading to possible further work up being required.

Lastly, it is presumed that concerning ECGs would warrant further work up without the context of the clinical situation for each individual ECG. A prospective study should be conducted next in which clinical scenarios along with concerning ECGs would warrant further work up to obtain a more accurate representation of the financial burden of this study center.

## Conclusions

Precordial lead mispositioning errors can lead to significantly abnormal ECG patterns which are then interpreted with consequential diagnoses. Therefore, substantial valid concern remains for general practitioners and primary care providers to suspect these abnormal patterns to possibly represent underlying CAD. This suspicion of CAD then leads to further downstream cardiovascular consultation and testing. Simple and minor errors of precordial ECG lead mispositioning probably cost us billions of dollars annually. In the current era of healthcare budgetary constraints and reforms demanding value healthcare, we need to focus on some of the root causes of wastage of healthcare dollars. Further rigorous and focused studies are needed to more accurately assess the financial burden of precordial ECG lead mispositioning in the US. Only then we would be able to address the root cause of the problem and develop strategic policies to curb healthcare dollar waste.
